# Community notes reduce engagement with and diffusion of false information online

**DOI:** 10.1073/pnas.2503413122

**Published:** 2025-09-18

**Authors:** Isaac Slaughter, Axel Peytavin, Johan Ugander, Martin Saveski

**Affiliations:** ^a^Information School, University of Washington, Seattle, WA 98195; ^b^Department of Management Science and Engineering, Stanford University, Stanford, CA 94305; ^c^Department of Statistics and Data Science, Yale University, New Haven, CT 06511

**Keywords:** misinformation, information diffusion, crowd-sourced fact-checking

## Abstract

Social networks scaffold the diffusion of information on social media. Much attention has been given to the spread of true vs. false content on online social platforms, including the structural differences between their diffusion patterns. However, much less is known about how platform interventions on false content alter the engagement with and diffusion of such content. In this work, we estimate the causal effects of Community Notes, a novel fact-checking feature adopted by X (formerly Twitter) to solicit and vet crowd-sourced fact-checking notes for false content. We gather detailed time series data for 40,078 posts for which notes have been proposed and use synthetic control methods to estimate a range of counterfactual outcomes. We find that attaching fact-checking notes significantly reduces the engagement with and diffusion of false content. We estimate that, on average, the notes resulted in reductions of 46.1% in reposts, 44.1% in likes, 21.9% in replies, and 13.5% in views after being attached. Over the posts’ entire lifespans, these reductions amount to 11.6% fewer reposts, 13.3% fewer likes, 6.9% fewer replies, and 5.5% fewer views on average. In reducing reposts, we observe that diffusion cascades for fact-checked content are less deep and less “viral,” but not less broad, than synthetic control estimates for non-fact-checked content with similar reach. This structural difference contrasts notably with differences between false vs. true content diffusion itself, where false information diffuses farther, but with structural patterns that are otherwise indistinguishable from those of true information, conditional on reach.

## Significance

Warning labels about misinformation in social media posts are typically provided by professional fact-checkers. Recently, X introduced Community Notes, a feature enabling ordinary users to propose and vet fact-checking notes for potentially misleading posts. We examine the impact of these fact-checking notes on how users interact with noted posts. We find that once a note is attached, posts receive significantly fewer reposts, likes, replies, and views. We also observe significant changes in the statistical properties of the network paths noted posts take, indicating that the intervention alters how they diffuse within the social network. These results suggest that crowd-sourced fact-checking can be an effective tool for mitigating misinformation online, providing a valuable addition to efforts to combat its spread.

The spread of false information on social media poses risks to public health (1), democratic processes (2), and social cohesion (3). Social media has been broadly observed to preferentially support the spread of false news over true news (4–7). Scholars as well as social media platforms are actively working to design and test strategies to limit its transmission (8–10), including fact-check warning labels placed on individual sources or pieces of information (11, 12), educational interventions to boost users’ competencies at identifying false information (13–16), and a shift to design objectives other than user engagement (17–20).

Professional fact-checking is the most widely used intervention against misinformation, often implemented by attaching warning labels to fact-checked posts (21–23). Studies investigating the effectiveness of these labels find that they decrease self-reported belief in and willingness to share misinformation (24–26). However, even if effective, professional fact-checking is costly and difficult to scale both in speed and coverage (27), and increasingly viewed with skepticism by segments of the public (28). Crowd-sourced fact-checking has emerged as a promising alternative, leveraging the “wisdom of the crowd,” i.e., that aggregating judgments of groups of nonexperts leads to accurate assessments even if the individual assessments are inaccurate (29). Lab experiments investigating the feasibility of crowd-sourced fact-checking find that groups as small as 15 people can identify misinformation as accurately as professional fact-checkers (30–33).

Building on these findings, X (formerly Twitter) introduced a crowd-sourced fact-checking system called Community Notes (34). The system enables ordinary users to propose fact-checking notes to be attached to potentially misleading posts and rate the helpfulness of proposed notes. The system uses a “bridging-based” matrix factorization algorithm to score the overall helpfulness of notes based on the individual ratings (35). Notes rated helpful by many users with diverse views, as measured by estimated latent positions, are scored higher. Only notes that cross a certain helpfulness threshold are classified helpful and displayed with the post.

Upon introducing the Community Notes program, X reported results from an A/B test that notes selected by the bridging-based algorithm reduced individual-level decisions to like and repost misinformation by 25 to 34% relative to a control group (35). Through the lens of widely employed epidemiological models of information diffusion, changes in the probability that individual units will share content typically have a highly nonlinear relationship with the overall number of people exposed to the content (36), a quantity that is not easily assessed through an A/B test due to ubiquitous network effects, i.e., interference between treatment and control units (37).

In this work, we investigate the causal effects of attaching community notes to posts on the engagement with and diffusion of the posts. We collect time-series data for 40,078 posts created between March and June 2023 for which community notes were proposed. We track key engagement metrics of the posts, including the number of reposts, likes, replies, and views over time. We also collect all reposts since the post was created, both before and after a note was proposed, and the follow graphs of the users that reposted them, which we use to reconstruct the diffusion cascades of the posts. These granular records concerning a post’s engagement both before and after a community note appears allow us to provide precise estimates of the notes’ effects, and investigate the conditions under which community notes are more or less successful at reducing the impact of misinformation.

**Fig. 1.**
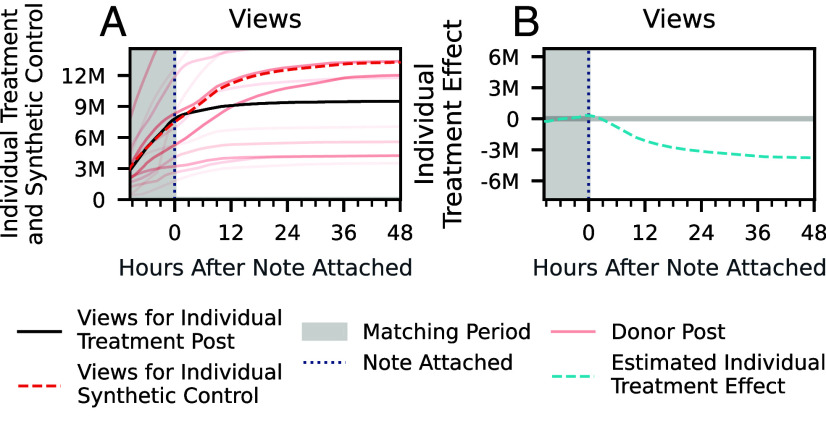
Illustration of synthetic control methodology for views. (*A*) Total number of views over time before and after receiving a community note for a sample post. The black line represents the post’s observed view count, and the dashed red line depicts the synthetic control’s view count, an estimate of what would have occurred had no note been attached. Individual donor posts that contribute to the synthetic control are shown in solid red lines with intensity of color proportional to their weight. Note that in addition to view counts, the synthetic control also has a similar trajectory on all other engagement and diffusion metrics. (*B*) Estimated difference between views of the synthetic control without the note and observed views with the note, quantifying the reduction in views attributable to note attachment at a given time.

We use synthetic control methods to estimate these causal effects (38, 39). For each post with a note attached, we construct a synthetic control by averaging the engagement histories of multiple donor posts—those for whom a note was proposed, but not attached—such that the synthetic control closely matches the history of all metrics of the noted post during the period before note attachment. Then, we estimate the effect of attaching a note by comparing engagement metrics during the period after the note was attached between the post and its synthetic control. This analysis produces an individual treatment effect for each post where a community note was attached. Fig. 1 illustrates the procedure for a sample post: Fig. 1*A* shows the number of views over time for the community noted post along with its estimated views had the note not been attached, while Fig. 1*B* shows the estimated treatment effect of the community note over time. To validate our estimation approach, we conduct an in-time placebo test (40) by artificially shifting the note attachment time one hour earlier and, as expected, estimate null effects in the period between the artificial and actual attachment time (further details can be found in *SI Appendix*, section 5).

We find that notes significantly reduce the number of views, reposts, likes, and replies. We interpret the impact on these metrics through two perspectives: a growth perspective, which quantifies the reduction in the additional growth of the metric after a note was attached, and an overall perspective, which quantifies the total reduction in the metric since the post’s creation. The growth perspective measures effectiveness conditional on when the note was attached, while the overall perspective also accounts for the engagement that occurred before the note was attached. These varied perspectives are related to measures of the “preventable fraction among the unexposed” and “preventable fraction” in epidemiology (41).

Beyond engagement, we also consider the impact of note attachment on the structure of information diffusion. Previous studies have found that fact-checked false news has larger, deeper, wider, and more viral diffusion cascades than fact-checked true news (5). Subsequent analyses of the same data have shown that while there are significant differences in cascade size, the structural differences disappear after controlling for size, suggesting that mechanisms through which true and false fact-checked news diffuse are relatively similar (6). In our setting, we find that note attachment qualitatively changes the structure of the post’s diffusion cascade, relative to the same post without a note. Most significantly, it reduces the depth and structural virality more than would be expected given the overall reduction in size.

Our rich data on diverse engagement metrics (reposts, replies, likes, and views) as well as our reconstruction of diffusion cascades (enabling us to study how note attachment influences cascade structure) go beyond earlier work studying the effects of Community Notes (42), which considered only the effects on reposts and deletions. That prior work also used difference-in-differences methods which, unlike our synthetic control methods, rely on strong “parallel trends” assumptions (43). When comparable, our independent estimates also provide important corroboration of those prior estimated effects.

Since our synthetic control methods approach provides causal effect estimates at the individual post level, we can examine how average effects vary across different post subpopulations. Overall, we find that notes have the greatest absolute impact on reducing engagement when they are attached shortly after a post is created or attached to highly engaging posts. We find that notes on posts with embedded media, as opposed to text-only posts, are associated with larger reductions. We also see larger reductions on posts where concerns about altered media are presented as a reason for the note. In terms of differences across how notes are composed, we find that moderately long notes and notes written using simpler language are associated with larger reductions in engagement.

## Data Collection

We collected data from March 16 to June 23, 2023, tracking 40,078 posts for which a note was proposed. We continuously monitored the “New” tab of the Community Notes website, which provides the identifiers of the posts for which notes were recently proposed. When a community note was created, we immediately retrieved the associated post’s engagement metrics using the X API. We then made API calls every five minutes to record the post’s total number of reposts, likes, replies, and views for three weeks following note creation. To ensure that all post engagement histories are comparable when constructing synthetic control weights, we shifted the engagement metrics to a timeline aligned with post creation rather than note creation time by linearly interpolating to fifteen-minute intervals. All engagement and diffusion metrics are all-cause measures, regardless of whether the content was delivered through an algorithmic or reverse-chronological feed.

We use public data available from X to determine when notes were attached to posts and which notes were never attached to any posts. Among the 40,078 posts included for analysis, 6,757 (16.9%) received helpful notes and constituted the treatment group. (Further details on the construction of the treatment group are provided in *SI Appendix*, section 1B.) The remaining 33,321 posts, for which a note was proposed but no note reached a helpful status, and thus no intervention took place, constitute the donor pool for constructing our synthetic controls. We focus our analysis on the effects of community notes within a 48-h window after a note is attached. Given the rapid decay of engagement on X (44), our 48-h estimates closely approximate the lifetime effects for posts that remain on the platform.

In addition to collecting engagement metrics starting when a first community note was written about a post, we also collected each post’s full public repost and reply history, extending back to its creation. However, we were unable to collect these data for deleted and private posts. In such cases, we relied on the repost and reply counts returned by the X API. We collected full public repost histories for 36,408 posts (90.8%) and full public reply histories for 30,858 posts (77.0%). We provide further details on the data cleaning and cascade data collection in *SI Appendix*, section 1.

## Results

### Decline in Average Engagement.

**Fig. 2.**
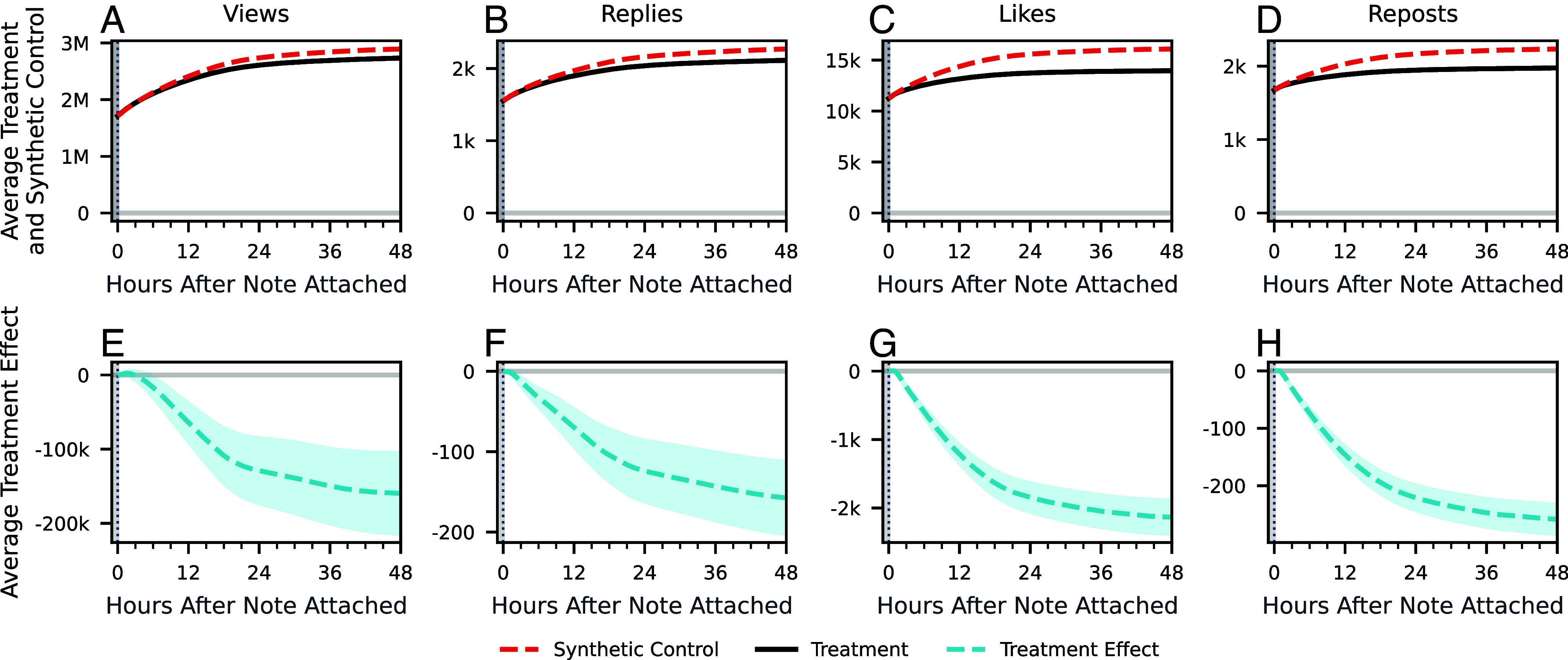
Effects of note attachment on views, replies, likes, and reposts. (*A*–*D*) Average treatment and synthetic control for all engagement metrics. Average treatment consists of the average value for a metric at a given time point among posts that received notes. Average synthetic control consists of the average value of the synthetic controls, estimating what the average would have been had notes not been attached. (*E*–*H*) Average treatment effect on the treated for all engagement metrics. Estimates the average difference between the treatment and synthetic control, i.e., the average reduction in a metric due to note attachment. The error bands represent 95% CIs.

We use synthetic control methods to estimate individual treatment effects on the number of views, replies, likes, and reposts for each post with a note reaching helpful status. We then aggregate these effects into average treatment effects on the treated population. Details on the construction of synthetic controls and the uncertainty quantification underlying the CIs can be found in *Materials and Methods*. Fig. 2*A* shows the average number of views over the 48 h after a note was attached for posts that received community notes, along with the average number of views for the same posts’ synthetic controls. Fig. 2*E* shows the average treatment effect on the treated, i.e., the average treatment effect across all noted posts. The equivalent figures for the number of replies, likes, and reposts are shown in Figs. 2 *B*–*D* and *F*–*H*, respectively. These measures quantify the aggregate impact of the Community Notes program. We discuss the heterogeneities among posts in *Factors Associated with Large Effects* and the distribution of individual treatment effects in *SI Appendix*, section 2.

We first discuss the aggregate effect of attaching notes on the posts’ number of views. We estimate that the average number of views for posts that received helpful notes decreased from 2.89 million views (95% CI: [2.84M, 2.95M]) to 2.73 million views due to note attachment. These estimates represent the total number of views that noted posts received, or would have received without notes, 48 h after they had community notes attached. The estimated treatment effect at this time amounts to −159,592 views (95% CI: [−214,839, −104,344]). This corresponds to a −13.5% reduction in additional growth in the number of views after note attachment or a −5.5% reduction in the total number of views, including those that occurred before the note was attached. Note that the first metric captures how the presence of a note affects a post’s ability to gain new views, while the second measures the overall impact of community notes at the platform level.

While the decrease in views reflects how community notes limit the posts’ reach, change in engagement metrics that require active participation from the users—specifically replies, likes, and reposts—capture how the notes affect the way users interact with the posts. Replies on social media may signal some combination of agreement, disagreement, or confusion from a replier. We estimate that the average number of replies to posts receiving helpful notes decreased by a similar percentage as the number of views: from 2,270 replies (95% CI: [2,225, 2,315]) to 2,112, a change of −158 replies (95% CI: [-203, −112]), which amounts to a −21.9% change in reply growth after attachment or −6.9% change in total number of replies.

Likes and reposts, on the other hand, are more frequently used as signals of positive engagement: likes on social media can indicate that the user finds the post enjoyable, useful, or interesting (45, 46), while reposts can signal agreement or serve to amplify a message (47). We estimate that the average number of likes given to posts that received helpful notes during this period fell from 16,089 (95% CI: [15,839, 16,340]) to 13,955 due to note attachment, an absolute change of −2,134 likes (95% CI: [−2,385, −1,884]). This amounts to a change of −44.1% in likes after note attachment and a change of −13.3% in total likes. Similarly, relative to the average synthetic control of 2,234 (95% CI: [2,207, 2,260]), we estimate that note attachment led to a change of −259 reposts (95% CI: [−285, −232]), bringing the observed average down to 1,975. This amounts to a percentage change in reposts after attachment of −46.1% and a percentage change in total reposts of −11.6%.

### Altered Dynamics of Information Diffusion.

Having found that community notes lead to sizable reductions in average engagement, we next examine their impact on how information spreads on the platform, specifically their effect on the structure of repost cascades. A repost cascade records the tree of reposts stemming from a post. The max depth of a cascade refers to the length of the longest chain of reposts it contains, max breadth refers to the maximum number of reposts at any level of depth, and structural virality refers to the average distance between any two nodes in the cascade, standardized by its size (48). A cascade with high breadth or low structural virality suggests that the post spread primarily through direct reposts of the original post. In contrast, high depth or high structural virality indicates the post spread more through multistep, person-to-person reposting chains, a pattern often seen with rumors and viral content (48). Additional details on the calculation of these metrics can be found in *SI Appendix*, section 1E.

**Fig. 3.**
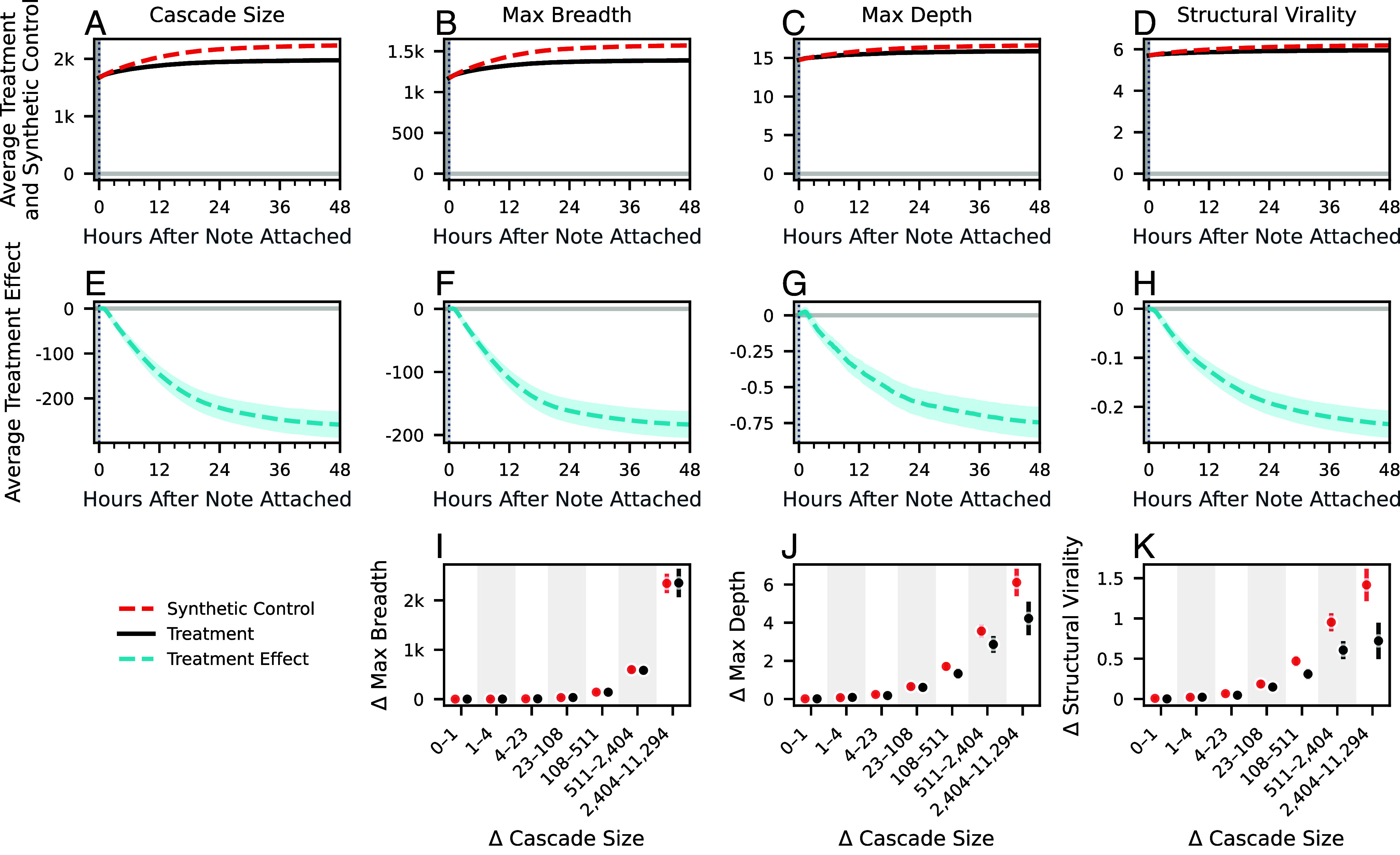
Effects of note attachment on the structure of repost cascades. (*A*–*D*) Average treatment and synthetic control values for (*A*) the size of the repost cascade (equivalent to the total number of reposts), (*B*) the maximum breadth of the repost cascade (which frequently occurs at the first level of the cascade and is, hence, a proxy for the number of direct reposts), (*C*) the maximum depth of the repost cascade (the longest chain of person-to-person reposting), and (*D*) the structural virality of the repost cascade (intended to measure the extent to which the post spread “virally,” where larger numbers indicate more viral diffusion). (*E*–*H*) Average treatment effect on the treated posts for all structural metrics. (*I*–*K*) Change in cascade structure controlling for the growth in cascade size after note attachment. For individual treatment posts and synthetic controls whose repost cascades grew by a given amount in the 48 h between t=0 and t=48, the plot shows the binned average growth in a structural metric over that same period. The bin edges evenly divide the positive range of Δ Cascade Size in logarithmic scale. The error bands and error bars represent 95% CIs.

As before, we construct a synthetic control for each post that received a helpful note, but now estimate treatment effects for structural metrics that characterize the post’s repost cascade: its max breadth, max depth, and structural virality. We again take the mean of these synthetic controls, as shown in Fig. 3*A*–*D*, along with the average observed values under treatment, and calculate average treatment effects on the treated, shown in Fig. 3*E*–*H*. The effect on the total number of reposts, also referred to as cascade size, is repeated in Fig. 3*A* and Fig. 3*E* for reference.

As with engagement metrics, we estimate that note attachment leads to a reduction in all structural metrics of the repost cascade after 48 h, relative to what would have been expected had the note not been attached. The average max breadth of synthetic controls is 1,571 reposts (95% CI: [1,552, 1,590]), while the observed value under treatment was 1,388 reposts, producing an estimated change of −183 reposts (95% CI: [−202, −165]) or −46.0% in growth after note attachment, or −11.7% in total. The estimated average max depth under control is 16.61 reposts (95% CI: [16.51, 16.71]), compared to the observed average of 15.87 reposts, a treatment effect of −0.74 reposts (95% CI: [−0.84, −0.65]), or −39.9% growth after note attachment, or −4.5% change in total. Finally, the average structural virality under control is estimated to be 6.19 (95% CI: [6.16, 6.21]) while the observed value is 5.95, a change of −0.24 absolute units (95% CI: [−0.26, −0.21]), or −48.5% growth after note attachment, or −3.9% in total.

Smaller repost cascades tend to be both less broad and less deep than larger repost cascades (6). To disentangle changes in size from changes in structural metrics, we perform the following matching procedure. For each post that received a note, we first calculate the growth in observed cascade size as well as in the observed structural metrics in the 48 h after note attachment. We apply the same procedure to the estimated synthetic controls. Finally, we compare the change in structural metrics for treated posts and synthetic controls whose repost cascades grew in size by a similar amount after note attachment. As shown in Fig. 3*I*–*K*, we find that while max breadth does not differ in the distribution between treatment and synthetic controls that grow by similar amounts, max depth and structural virality do differ. Relative to synthetic controls that grew by a similar amount, posts that receive notes do so with a smaller max depth and smaller structural virality, indicating less viral diffusion in the presence of an attached note. These results suggest that effects of attaching a community note on depth and structural virality cannot be simply explained by a change in cascade size and suggest that the attachment of a note significantly affects the mechanism through which the posts spread over the network. These structural differences are consistent with community notes having a larger moderating effect on users when the post reaches them through a repost cascade, and less of an effect on users who receive the post directly from the original poster.

### Factors Associated with Large Effects.

Our previous analyses show that community notes significantly reduce the average engagement with misleading posts and change the diffusion patterns of such posts. However, their effects are not uniform across all types of posts or notes. Next, we perform exploratory analyses to identify the contexts in which attaching community notes has the largest impact. We caution that the results presented below are associations and should not be interpreted as causal. While our synthetic controls methodology enables us to estimate the causal effects of attaching a community note to an individual post, the associations between the post or note factors and the estimated effects may be confounded by other observed and unobserved characteristics. We consider estimating the causal impact of the factors most strongly associated with large effects an important direction for future work.

**Fig. 4.**
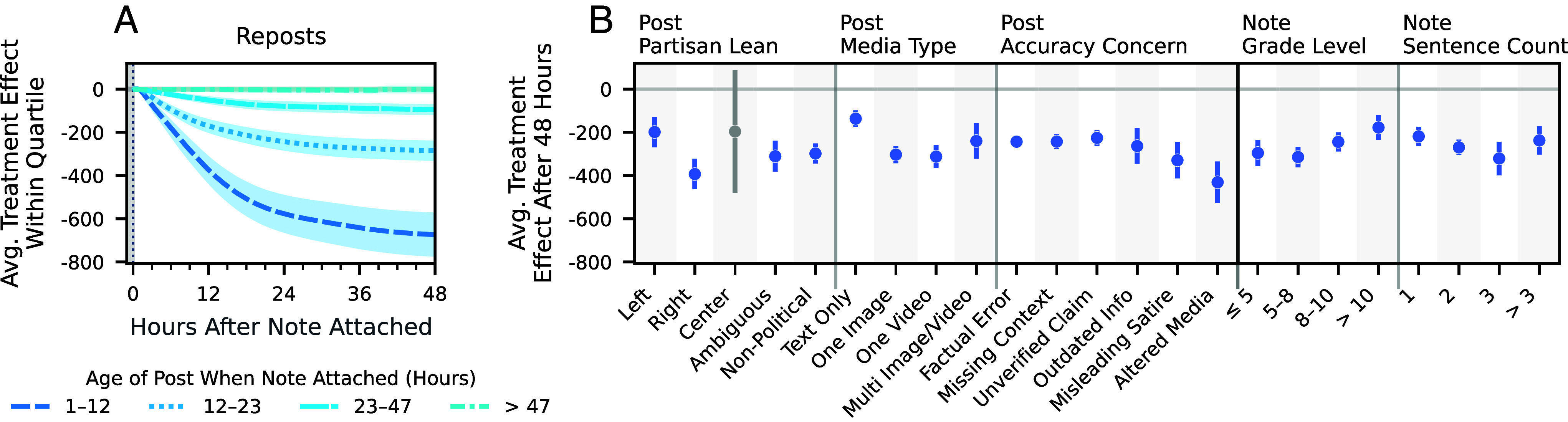
Factors associated with effects on reposts. (*A*) Average treatment effects for noted posts, stratified by quartiles of note attachment speed, i.e., the time elapsed between the post’s creation and note attachment. (*B*) Average treatment effects for noted posts after 48 h, based on i) the post’s partisan lean, ii) the number of images and videos, iii) the accuracy concerns raised by the community note writer, iv) the readability (Flesch-Kincaid grade level), and v) the length of the community note. The error bands and error bars represent 95% CIs.

In Fig. 4*A*, we show the average treatment effect of community notes on reposts, stratified by speed of note attachment. We find that notes attached soon after a post is created are more effective at reducing reposts. Specifically, the absolute estimated treatment effects for the first quartile (attached within 1 to 12 h), second quartile (12 to 23 h), third quartile (23 to 47 h), and fourth quartile (47+ h) are −673 (95% CI: [−768, −579]), −285 (95% CI: [−325, −246]), −95 (95% CI: [−113, −77]), and −2 (95% CI: [−13, 8]), respectively. The corresponding percentage reductions in repost growth following note attachment are −49.6%, −44.1%, −38.8%, and −6.2%, while the total repost reductions amount to −24.9%, −12.3%, −4.3%, and −0.1%, respectively.

As shown in *SI Appendix*, section 4, other engagement metrics exhibit similar monotonically decreasing effects when stratified by speed of note attachment. One nuance is that for posts in the fourth quartile (47+ h from post creation to note attachment), the percentage changes in average growth of views and replies are positive: an increase of 13.6% for views and an increase of 27.0% for replies 48 h after note attachment. This increase suggests that community notes may draw attention to stale posts. However, it does not imply that the notes draw more endorsement or agreement, as the average growth for likes and reposts (as opposed to views) within this quartile are −22.2% and −6.2%, respectively.

Another major factor associated with the magnitude of the treatment effect is the volume of engagement a post received before a note was attached: posts that received more reposts prior to note attachment have larger treatment effects on average. In *SI Appendix*, section 3, we show that while the most popular posts exhibit larger absolute drops in reposts after note attachment, their percentage changes are relatively similar, suggesting these declines stem mostly from their larger baseline audience. In contrast, posts with low initial repost counts often experience small positive changes, indicating that note attachment can sometimes boost the visibility of less prominent content.

While the majority of variation in treatment effects is attributable to the speed of note attachment and the posts’ popularity before treatment, we also find that both post content and note quality are associated with variability in treatment effects. Prior work has found that right-leaning media consumers tend to be more prone to motivated reasoning than left-leaning consumers (49) and that Republicans are less likely than Democrats to rate community notes as helpful (35). These findings suggest that notes on right-leaning content, likely seen more often by right-leaning consumers (50), would have smaller treatment effects than those on left-leaning content. However, we do not find evidence that this is the case among the English-language posts that we label for partisanship. In fact, we estimate that notes on left-leaning content tend to be less effective at reducing reposts, both in absolute and relative terms. We describe the methodology for labeling post partisanship in *SI Appendix*, section 1F. The estimated average treatment effect on right-leaning posts is −393 reposts (95% CI: [−464, −323]), a percentage change in average growth due to note attachment of −55.1%, relative to an estimated average treatment effect on left-leaning posts of −198 reposts (95% CI: [−269, −128]), a percentage change of −41.8%. As shown in *SI Appendix*, section 4, we also find similar effects for views, replies, and likes.

Beyond partisanship, we also find that the type of media included in the post is associated with note effectiveness. The estimated treatment effect for media posts—those containing a single image, single video, or multiple images/videos—are −303 reposts (95% CI: [−343, −263]), −312 reposts (95% CI: [−365, −260]), and −240 reposts (95% CI: [−322, −158]), respectively. In contrast, text-only posts have an estimated treatment effect of −137 (95% CI: [−175, −98]). While media posts generally receive more engagement than text-only posts before receiving a note, we find that the difference in average effect persists even after normalizing by size. The estimated percent changes in average growth due to note attachment are −50.7%, −45.6%, −48.7% for a single image, single video, and multi-image/video posts, respectively, compared to −37.9% for text-only posts.

The sizes of the treatment effects also vary depending on what type of concern the community note addresses. When proposing a community note, writers are asked to indicate their accuracy concern, i.e., what aspect of the post they consider misleading. They can select multiple concerns, e.g., that the post both contains outdated information and makes a factual error. As shown in Fig. 4*B*, we find that concerns related to altered media and misleading satire are most strongly associated with large effects.

Considering the community notes themselves, we find that both readability and length are associated with effectiveness. We measure readability using the Flesch-Kincaid grade level score, an estimate of the minimum U.S. grade level required to comprehend a text (51). We find that simpler notes tend to be more effective. Notes with grade levels less than or equal to five have an estimated average treatment effect of −296 reposts (95% CI: [−356, −235]), corresponding to a −47.4% change in average growth after note attachment. In contrast, notes with a grade level above ten have an estimated average treatment effect of −178 reposts (95% CI: [−234, −121]), which amounts to a change of −38.8%. Finally, we find that moderately long notes (two or three sentences) are slightly more effective than both short (one sentence) and long (more than three sentences) notes at reducing reposts.

## Discussion

As the problem of misinformation persists on social media, scalable interventions are necessary to prevent its spread, impact, and harm. Crowd-sourced fact-checking presents one such approach, which has demonstrated encouraging results in early tests and is now deployed as a core content moderation component on one of the world’s largest social media platforms. The public nature of Community Notes’ deployment on X, with freely available source code and rating data, as well as detailed information on the content and engagement of posts over time, has allowed us to estimate the impact of community-driven fact-checking through a completely independent audit.

Our results indicate that once community notes are attached, on average, they reduce the engagement with and diffusion of false information on X. Consistent with related work by Chuai et al. (42), which studied reposts and deletions, we find that community notes lead to a decline in the number of reposts that a post receives after attachment. Despite differences in causal identification strategies, our estimate of percentage decline in average reposts after note attachment, −46.1% during the period March 16 to June 23, 2023, is roughly comparable with estimates from Chuai et al. of −55.2%, −49.6%, −45.6%, and −47.5% in the months of March, April, May, and June of 2023. Our analysis of other outcomes finds that notes lead to a similar percentage reduction in likes (−44.1%) but smaller reductions in views (−13.5%) and replies (−21.9%).

These findings suggest that the impact of note attachment is strongest on public expressions of support for content (reposts and likes), while its effect is smaller on whether content reaches people in the first place (views) or whether they choose to engage in an online conversation about it (replies). While these differences might be taken to imply that the reduced support nullifies the effects of misinformation exposure after attachment, we caution against this interpretation. Viewing false information, even if the viewer initially doubts its validity, can increase their likelihood of agreeing with it later (52). Thus, each view prevented by a community note is meaningful. We also note that the decline in reposts and likes may not necessarily reflect a decline in actual support of the content but rather a reduced willingness among users to signal their support publicly. An internal analysis by X does report that users are less likely to agree with the substance of potentially misleading posts when presented with a community note (35).

The decline in engagement is paired with notable structural changes in how posts diffuse across the platform. The changes are consistent with larger behavioral changes by users who do not follow the original post’s author. Such larger changes can be attributed to homophily (53), where users closer to the root author may share similar beliefs or information evaluation approaches with the root author. As an alternative mechanism, these results are also consistent with dyadic social pressures (47) whereby users may feel greater loyalty obligations to close connections than those encountered through deeper network paths.

While we find that community notes effectively reduce engagement once attached (−13.5% views, −21.9% replies, −44.1% likes, and −46.1% reposts), we also find evidence that the system would be much more effective if notes were attached faster. Moreover, the reductions in views, replies, likes, and reposts are much more modest when measured as a percentage of overall engagement with the post, compared to only considering changes in engagement after note attachment. When measured this way, the overall percentage changes in views, replies, likes, and reposts due to note attachment are −5.5%, −6.9%, −13.3%, and −11.6%, respectively.

Our study has several limitations worth considering. First, like most observational studies, our analysis relies on certain assumptions to identify causal effects. To examine the plausibility of these assumptions, we perform a series of robustness checks, including i) a placebo test, ii) limiting the donor pool for constructing synthetic controls to posts with notes that received high scores from the Community Notes algorithm, and iii) incorporating semantic embeddings of the posts—alongside engagement and diffusion trajectories—when constructing synthetic controls. The results of the placebo test were consistent with suitable controls being in place (*SI Appendix*, section 5), and neither limiting the donor pool nor incorporating the posts’ semantic embeddings into the construction of the synthetic controls substantively changed the conclusions of our analysis (*SI Appendix*, sections 7 and 8). Second, our analysis can only estimate the effects of community notes on posts that had notes attached. Based on the data available to us, we cannot estimate the coverage of the Community Notes program (i.e., how many misleading posts on X do or do not received a note) or what the effectiveness of notes might be on the broader population of posts without notes. Such analysis is inherently challenging as it requires both access to all posts on the platform and a scalable method for identifying misleading posts. Third, we cannot test for implied truth effects (54), i.e., the potentially increased tendency for posts without community notes to be perceived as accurate or nonmisleading, even when they are not. Finally, Community Notes is an evolving system and our analysis reflects the effects of the system during the study period, March–June 2023. Like all social media research, the rapidly changing environment makes temporal validity challenging (55). Since we concluded our data collection, more volunteers have joined the program, extensions of the system have been proposed (56), and substantial updates to the system’s implementation have been introduced. These updates include improvements in the time required to run the algorithm and display the notes (57), as well as the automatic attachment of a community note to posts with images and links that were previously included in other noted posts. Our findings suggest that these changes are likely to lead to significant additional reductions in engagement with misleading content on X. Nevertheless, community-based fact-checking is best viewed as one of several interventions (9) worth considering when aiming to reduce the spread of misinformation on social media.

## Materials and Methods

### Estimating Individual Treatment Effects.

We use synthetic control methods to estimate the effect of receiving a community note on a post’s engagement and diffusion. These methods are commonly employed to estimate causal effects when detailed time-series data is available for both treated and untreated units (38, 39). Under the synthetic controls framework, each unit that receives the intervention (in our case, note attachment, which we also refer to as “treatment”) receives an individual synthetic control estimate, which is interpreted as what would have happened to the treated unit had the intervention not occurred (i.e., had it never received a community note). Observations of the outcome for treatment unit i are denoted as $Y_{\mathrm{imt}}\left(Z_{i}=1\right)$, where m refers to the metric in question (e.g., reposts), t refers to the amount of time elapsed since note attachment (e.g., 48 h after attachment), and the value of $Z_{i}=1$ indicates that we are referring to unit i when it received treatment. We refer to estimates from the synthetic control as ${\hat{Y}}_{\mathrm{imt}}\left(Z_{i}=0\right)$.

A synthetic control is a weighted average of donor posts: ${\hat{Y}}_{\mathrm{imt}}\left(0\right)=\sum_{j\in D_{i}}w_{\mathrm{ij}}Y_{\mathrm{jmt}}\left(0\right)$, where $D_{i}$ is the set of eligible donors for post i and $w_{\mathrm{ij}}$ are non-negative weights. As donors, we considered posts for which at least one community note was proposed, but no note was ever found to be helpful, and which therefore never experienced note attachment. For each treated post, we further restricted the donor pool to untreated posts for which historical data were available in the time period around which the treated post received a helpful note. For treated posts with more than 12 h of historical data, we used only the 12 h immediately preceding treatment. This choice allowed us to avoid requiring donor posts to always have histories as long as, or longer than, those of the treated posts, thus helping us maintain a suitable number of donors for each treated post. To ensure a sufficient amount of data for constructing estimates for each individual metric, we considered only treated posts with at least one hour of historical data prior to note attachment for the given metric.

To determine the weights for each synthetic control, we minimized the squared Euclidean distance between the metrics of the treated post and those of its synthetic control, using data up to the time point when the treated post had a note attached: $\sum_{\left(m,t\right)\in M_{i}\times T_{i}:t<0}[Y_{\mathrm{imt}}\left(1\right)-{\hat{Y}}_{\mathrm{imt}}\left(0\right){]}^{2}$, where $M_{i}$ refers to the complete set of metrics for unit i, and $T_{i}$ refers to the complete set of time points. We construct weights by minimizing this distance across the following metrics, when available for a post: views, replies, likes, reposts, author follower count, repost cascade maximum breadth, repost cascade maximum depth, and repost cascade structural virality (*SI Appendix*, section 1D). In total, inferring the weights required solving 6,757 linearly constrained least squares problems (one for each treated post), each of which was a quadratic program. Due to the computational burden of solving each of these programs, we restrict the donor pool for each treated post to the 1,000 control posts closest to that treated unit in Euclidean distance. This restriction greatly reduces the computational cost of solving each quadratic program and can be viewed as a hard thresholding analog of the “penalized” synthetic control method (39). To prevent metrics with larger scales (e.g., views) from dominating the synthetic control construction, we standardize all metrics by their sample SD within the treated posts, following Abadie and L’Hour (39).

We refer to the true individual treatment effect for unit i, metric m, at time point t as $\tau_{\mathrm{imt}}=Y_{\mathrm{imt}}\left(1\right)-Y_{\mathrm{imt}}\left(0\right)$, and ${\hat{\tau }}_{\mathrm{imt}}$ as our estimate of that quantity using synthetic controls. One approach to estimating this effect would be to consider the simple difference between the treated post’s engagement and the corresponding engagement for its synthetic control, $Y_{\mathrm{imt}}\left(1\right)-{\hat{Y}}_{\mathrm{imt}}\left(0\right)$. However, this estimation method can induce bias in ${\hat{\tau }}_{\mathrm{imt}}$ if the treatment unit and synthetic control do not closely match prior to treatment, which is not always possible in high-dimensional datasets (58). We therefore employ the bias correction procedure recommended by Abadie and L’Hour (39) to address imperfect matches between treatment posts and their synthetic controls when estimating individual treatment effects. The procedure involves fitting regression models on untreated posts that predict all posttreatment outcomes (one model for each metric and time point combination) based on their pretreatment history. We fit ordinary least squares models. To build the training datasets, we sampled 100 donor posts from each treated unit’s donor pool and combined them. We then estimate the bias-corrected synthetic controls as ${\hat{Y}}_{\mathrm{imt}}^{\mathrm{BC}}\left(0\right)=\sum_{j\in D_{i}}w_{\mathrm{ij}}Y_{\mathrm{jmt}}\left(0\right)-\sum_{j\in D_{i}}w_{\mathrm{ij}}\left[{\hat{\mu }}_{\mathrm{mt}}^{\left(0\right)}\left(X_{i}\right)-{\hat{\mu }}_{\mathrm{mt}}^{\left(0\right)}\left(X_{j}\right)\right]$, where ${\hat{\mu }}_{\mathrm{mt}}^{\left(0\right)}$ is the model for metric m for posttreatment time period t, and $X_{i}$ and $X_{j}$ are the pretreatment covariates.

### Aggregating Individual Treatment Effects.

We calculate several statistics to summarize the estimated individual effects. First, we calculate the average estimated treatment effect on the treated posts as ${\hat{\tau }}_{\cdot mt}=\frac{1}{|N_{\cdot mt}|}\sum_{i\in N_{\cdot mt}}{\hat{\tau }}_{\mathrm{imt}}$ (shown in Figs. 2*E*–*H* and 3*E*–*H*), where $N_{\cdot mt}$ refers to the complete set of treated units for a metric m and time point t. Referring to the average observed value under treatment as $Y_{\cdot mt}\left(1\right)$, and the average estimated (bias-corrected) synthetic control value as ${\hat{Y}}_{\cdot mt}^{\mathrm{BC}}\left(0\right)$, then ${\hat{\tau }}_{\cdot mt}$ is equivalent to $Y_{\cdot mt}\left(1\right)-{\hat{Y}}_{\cdot mt}^{\mathrm{BC}}\left(0\right)$. This means that the average estimated treatment effect can be interpreted as the absolute change in average outcome due to note attachment.

To quantify the uncertainty of the synthetic control estimation procedure, we use standard 95% Gaussian CIs, defined as: ${\hat{\tau }}_{\cdot mt}\pm z_{\alpha /2}\frac{{\hat{\sigma }}_{\cdot mt}}{|N_{\cdot mt}|}$, where ${\hat{\sigma }}_{\cdot mt}$ denotes the estimated SD of the treatment effect for a given metric and time point. In the analyses of factors associated with large effects, we use the equivalent CIs, restricted to the relevant subpopulations. In addition to reporting CIs, we conduct a permutation test to further assess the statistical significance of our results (*SI Appendix*, section 6).

In addition to calculating absolute change, we also compute the percentage change in average outcome due to note attachment: $\frac{Y_{\cdot mt}\left(1\right)-{\hat{Y}}_{\cdot mt}^{\mathrm{BC}}\left(0\right)}{{\hat{Y}}_{\cdot mt}^{\mathrm{BC}}\left(0\right)}$. This metric normalizes by the average estimated outcome had notes never been attached to the posts considered, reflecting the overall impact of the Community Notes program. Importantly, it also accounts for the time required for notes to be written and rated as part of the treatment. To summarize the effect of notes once they have been attached, we also consider the growth after treatment: $\Delta Y_{\cdot mt}\left(1\right)=Y_{\cdot mt}\left(1\right)-Y_{\cdot m0}\left(1\right)$ in the treatment unit, along with the comparable growth in the control unit, $\Delta {\hat{Y}}_{\cdot mt}^{\mathrm{BC}}\left(0\right)={\hat{Y}}_{\cdot mt}^{\mathrm{BC}}\left(0\right)-{\hat{Y}}_{\cdot m0}^{\mathrm{BC}}\left(0\right)$. The percentage change in growth is then $\frac{\Delta Y_{\cdot mt}\left(1\right)-\Delta {\hat{Y}}_{\cdot mt}^{\mathrm{BC}}\left(0\right)}{\Delta {\hat{Y}}_{\cdot mt}^{\mathrm{BC}}\left(0\right)}$.

## Supplementary Material

Appendix 01 (PDF)

## Data Availability

Anonymized replication data is available in the Harvard Dataverse (59), and analysis code is available on GitHub (60).
