# Supplementary material for: Community notes reduce engagement with and diffusion of false information online
Source: Proc Natl Acad Sci U S A. 2025 Sep 18;122(38):e2503413122. doi: 10.1073/pnas.2503413122 (PMC12478135; doi:10.1073/pnas.2503413122)
Supplement: Supplementary file 1 — Appendix 01 (PDF) [file pnas.2503413122.sapp.pdf]

Supporting Information for

Community notes reduce engagement with and diffusion of false information online

Isaac Slaughter, Axel Peytavin, Johan Ugander, and Martin Saveski

Corresponding Author: Martin Saveski
E-mail: msaveski@uw.edu

This PDF file includes:

Supporting text
Figs. S1 to S10
Tables S1 to S2
SI References

Supporting Information Text

1. Additional Details Concerning Data Collection and Processing

A. Data Collection Pipeline. We collected data from March 16 to June 23, 2023. On June 23, 2023, the X Academic Research API was discontinued, and a new pricing structure was implemented, making further data collection prohibitively expensive. We initially collected data for $41,310$ posts that were the subject of community notes, using the `/2/tweets` X API endpoint to collect counts of views, replies, likes, and reposts over time. We started querying this endpoint soon after a note for a given post was proposed and appeared on the "New" tab of the Community Notes website, which provides the identifiers of posts for which notes were recently proposed. Since the "New" tab includes only posts that have been liked or reposted more than 100 times in total, we also scanned the most recently released publicly available Community Notes data every hour to ensure we did not miss any posts. We continued querying the `/2/tweets` endpoint every five minutes for the following three weeks.

During data processing, we identified anomalies in the engagement metrics returned by the API for 874 of these posts (2.1%). These anomalies consisted of sharp rises and declines in engagement metrics (usually in only a single engagement metric), which we hypothesize were due to data processing issues on the X backend. We removed posts exhibiting such anomalies from our dataset, resulting in the inclusion of $40,436$ posts after filtering. We provide more details concerning the identification of anomalous posts in Section *1.C*.

We applied two additional filters: posts needed at least one hour of data prior to note attachment to ensure reliable synthetic control construction, and at least 48 hours of data afterward to maintain consistency in the set of posts used to compute average treatment effects across time points. These criteria excluded 355 posts (0.9%), reducing the dataset size to $40,081$. Three treated posts were later excluded because the quadratic programs used to determine their synthetic control weights failed to converge, resulting in a final count of $40,078$.

In addition to collecting data through the `/2/tweets` endpoint, we also used the full-archive (`/2/tweets/search/all`), recent search (`/2/tweets/search/recent`), and the follow graph (`/2/users/:id/followers` and `/2/users/:id/following`) endpoints. These endpoints provide information on which accounts publicly reposted or replied to posts that received community notes, allowing us both to calculate the exact number of reposts and replies at any given time since the posts' creation and to construct the repost cascades. Since these counts were exact, we used them as our default repost and reply count metrics, falling back on the repost and reply counts returned by the `/2/tweets` endpoint when they were unavailable.

B. Defining Treatment Status. X runs the "bridging-based" matrix factorization algorithm that classifies community notes as "helpful" every hour. While the majority of notes classified as "helpful" remain "helpful" for the 48-hour period over which we analyze the effects of the notes, some notes lose their "helpful" status. In our analysis, if a post had a "helpful" note at any point, even if it was later reclassified as "needs more ratings" or "not helpful," we consider it part of the treatment group for its entire lifespan. Of the $6,757$ treated posts in our analysis, $5,366$ had a "helpful" note for the entire 48-hour period after first receiving one (79.4%), meaning that 20.6% of posts experienced at least one additional change in status.

Transitions from "helpful" to "needs more ratings" or "not helpful" tend to occur after many viewers have already seen a post. The median transition occurred 15.5 hours after posts were originally rated "helpful." In fact, 65.9% of views for posts with notes that lost "helpful" status occurred while the post had a "helpful" note. Together, these facts indicate that most views of treated posts occur when the posts have notes attached: among all treated posts, 93.4% of views in the 48 hours after note attachment occurred while the posts had "helpful" notes.

Only one note can be attached to a post at a time, and when multiple notes are classified as "helpful" for a given post, X randomly selects one to display. Of the $6,757$ treated posts, 852 had more than one note classified as "helpful" in the 48 hours after initial note attachment (12.6%). For posts with multiple "helpful" notes, we use the note that first received a "helpful" status to determine the treatment time. Considering all treated posts, there were a total of $7,756$ notes potentially shown with the $6,757$ treated posts in the 48 hours following the first note attachment.

Data was not available on which note was actually shown alongside a post at a given time. When calculating post-level statistics related to the note a post received—e.g., a note's reading grade level in *Main Text, Fig. 4*—we take a weighted average across the different notes that appeared with the post, with weights proportional to the amount of time each individual note was rated as "helpful" during the 48 hours after note attachment.

C. Anomalous Post Removal. While exploring our dataset, we identified several posts that we considered to be anomalous. These posts showed large rises and drops in a subset of their engagement metrics, while other metrics exhibited more gradual changes over the same time period. In most cases only a single engagement metric was affected. We did not believe the metrics returned by the X API accurately reflected the true engagement with these posts and therefore elected to remove them from the analysis.

To identify posts for removal, we began by plotting a random sample of 300 posts that showed more than a 1% rise or drop in at least one metric between API calls, which must have also amounted to at least 10 absolute units, a permissive heuristic that captured all posts identified as anomalous during initial checks. Two authors (I.S. and M.S.) each labeled the engagement metrics of 150 posts as either (i) accurate or (ii) likely erroneous and in need of removal. After discussing the labeling criteria, they conducted a second round of labeling on an additional random sample of 300 posts that exhibited a drop of 0.5% to 3% in a metric (amounting to 20 to 100 absolute units), combined with a rise of the same magnitude. The authors each labeled 200 of the 300 posts and gave the same label to 49 of the 50 overlapping records (Krippendorff's $\alpha = 0.96$, 95% CI: $[0.85, 1]$).

	Control		Treatment	
	Unavailable	Available	Unavailable	Available
Reposts	110	33,211	16	6,741
Replies	122	33,199	10	6,747
Likes	740	32,581	208	6,549
Views	1,664	31,657	373	6,384
Repost Cascade Depth	10,088	23,233	1,171	5,586
Repost Cascade Width	10,088	23,233	1,171	5,586
Repost Cascade Structural Virality	10,365	22,956	1,176	5,581

Table S1. Missing data by metric and treatment status. Number of posts with at least one observation available for a metric, compared to the number of posts for which no observations could be retrieved.

	Treatment				
	Fully Available	Dropped Pre Treatment	Dropped Post Treatment	Only Post Treatment	Unavailable
Reposts	6,416	49	261	15	16
Replies	6,631	1	6	109	10
Likes	4,959	70	375	1,145	208
Views	4,842	69	366	1,107	373
Repost Cascade Depth	4,264	407	915	0	1,171
Repost Cascade Width	4,264	407	915	0	1,171
Repost Cascade Wiener Index	4,259	405	898	19	1,176

Table S2. Amount of data available for treatment posts. For each metric, the number of posts that had observations (i) available for at least 48 hours after treatment, (ii) available at some point before treatment but dropped before treatment occurred, (iii) available at time points both before and after treatment but not for a full 48 hours, (iv) available only after treatment had already occurred, and (v) never available.

After labeling a total of 600 posts, we tested various criteria for identifying anomalous posts. We performed a grid search to identify combinations of percentage and absolute rises and drops that minimized mislabeling. From the grid search, we selected the thresholds that maximized the number of posts that were correctly removed, among solutions that did not incorrectly remove *any* posts from the labeled training data. The optimized thresholds were a rise of 25 units, amounting to at least 3%, and at another point in time, a drop of 25 units, amounting to at least 3%. We finally applied these thresholds to the complete dataset, removing a total of 874 posts.

D. Missing Data. As described above, in addition to the publicly released Community Notes data, we also collected data through multiple X API endpoints. While we were able to collect complete data for most posts, there were cases where we could retrieve data from one endpoint but not another, or where we could retrieve data from an endpoint but only for some metrics (e.g., replies, likes, and reposts present, but views missing). Since we requested data every five minutes and later linearly interpolated observations to 15-minute intervals from each post's creation time, this missing data mostly did not pose an issue for the analysis. However, for some posts, we were never able to retrieve data from certain endpoints, or we found that the data we did retrieve consistently lacked observations for a specific metric. We also found cases where a single endpoint would stop returning responses after a certain time, while other endpoints would continue returning responses. Similarly, we observed that some metrics would stop being returned at a given time, while others would not. Rather than removing these posts from the analysis, we decided to use only the metrics that were available. For example, for posts missing view observations, we did not include views in the construction of synthetic controls and did not estimate the treatment effects on views. In Tables *S1* and *S2*, we report the number of posts for which each metric was available.

E. Structural Characteristics of Repost Cascades. For a given post, we construct its repost cascade as a directed tree using time-inferred diffusion (1), a standard procedure for attributing diffusion pathways (2). The post itself is the root node, and each repost constitutes another node in the cascade. Each repost has a directed edge to the likely source through which the reposter was exposed to the post, according to the following procedure: For each repost, we scan the set of users the reposter follows to find the one who most recently shared the content. A directed edge is then added from the reposter's node to the followee's node. If no user the reposter follows has reposted the content previously, a directed edge is added to the root node.

The maximum depth of a repost cascade refers to the longest path between the original post and any individual repost in the cascade graph. The maximum breadth is defined as the largest number of reposts occurring at any single level of depth. In addition to depth and breadth, we also calculate the structural virality of a post's repost cascade, which is closely related to the Wiener index (3) and captures the extent to which a post diffused virally (person-to-person) versus through a large broadcast. Structural virality is calculated as the average distance between all pairs of nodes in the repost cascade, treated as undirected (1). We calculate the exact values of maximum depth, maximum breadth, and structural virality every 15 minutes

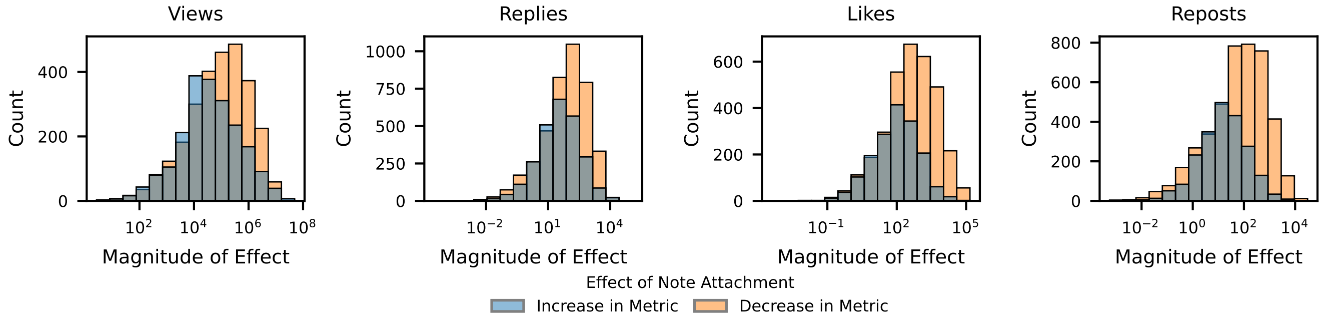

Fig. S1. Distribution of individual treatment effects on each engagement metric. Color indicates whether the treatment effect was positive (i.e., the note led to an increase in the metric) or negative, while the position on the x-axis (log scale) represents the magnitude of the increase or decrease. Plots show treatment effects after 48 hours.

after a post was created, based on the timestamps of reposts provided by the API. Since repost data was generally collected after the fact, deleted reposts are not included in the cascades. Reconstructing repost cascades requires data that we were not able to collect for all posts; as such, we only analyzed cascades for posts for which this data was available (Section *1.D*).

F. Post Partisanship Classification. We used the Claude 3.5-Sonnet V2 model ("claude-3-5-sonnet-v2@20241022") to label the partisanship of English-language posts. The prompt used was "Please annotate the tweet below using the following schema. It is okay if you cannot view photos, videos, or links from the tweet, but please do your best to interpret the text and any available context. Ensure that your response ends with a correctly formatted JSON containing all requested columns. Make sure that you do not include any other text after the JSON." A full JSON schema was provided, in which the description of the partisanship class was given as "The political leaning of the tweet content. The category 'unknown' indicates the political leaning is unclear, and 'none' indicates the content is not political." (In plots, "unknown" was changed to "ambiguous" and "none" was changed to "non-political" for clarity.) There were 904 posts labeled as "left," 1,360 posts labeled as "right," 53 posts labeled as "center," 691 posts labeled as "unknown," 1,908 labeled as "none," 1,580 non-English posts not labeled, and 261 posts not labeled due to unavailable post text.

2. Distribution of Treatment Effects

While we estimate that community notes result in declines in engagement on average, we find that for many posts, note attachment leads to *increased* engagement relative to their synthetic controls. We estimate that 43.0% of posts see increases in views after 48 hours due to note attachment, while the comparable number for replies is 39.2%, for likes is 33.9%, and for reposts is 33.0%. However, the estimated increases are typically smaller in magnitude than the decreases, resulting in the sizable negative average effects we observe. We plot the distributions of individual treatment effects for these four metrics in *Fig. S1*, showing increases and decreases separately. The plots show histograms of treatment effect magnitudes on a common log-scale axis, where the color indicates whether the treatment effect was positive or negative.

Considering positive and negative treatment effects separately, the average effects are 518,882 and $-671,893$ for views, respectively; 328 and -470 for replies; 799 and $-3,641$ for likes; and 120 and -445 for reposts. The medians show similar differences: the median positive treatment effect for views is 34,524, for example, about three times smaller in magnitude than the median negative treatment effect of $-109,949$. For replies, the medians are 39.3 and -97.8; for likes, the medians are 105 and -467; and for reposts, the medians are 16.4 and -75.5. (Medians are non-integer as synthetic control estimates need not be integers.) This analysis suggests that while community notes do at times result in increases in engagement, the increases tend to be both less frequent and smaller in magnitude than the decreases.

We state in the main text (*Decline in Average Engagement*) that the average declines in views and replies after note attachment are -13.5% and -21.9%, respectively, compared to -44.1% and -46.1% for likes and reposts. The difference between these sets of metrics suggests that community notes may have a stronger impact on engagement that clearly signals support for a post than on the number of people who simply view it or who engage with it in a more ambiguous way. The percentage of treatment effects that are positive (43.0% and 39.2% for views and replies, compared to 33.9% and 33.0% for likes and reposts) suggests that part of this effect is because community notes more often lead to increases in views and replies than in likes and reposts. The distributions of magnitudes for positive and negative effects suggest another dimension to the differences in averages. When comparing positive and negative treatment effects that come from the same percentile of the magnitude distribution, we find that for views and replies, the positive and negative treatment effects tend to be more similar to each other than the corresponding percentiles for likes and reposts. For example, the median negative views and replies treatment effects are 3.18 and 2.49 times as large as their positive counterparts, respectively. In comparison, the median negative likes and reposts treatment effects are 4.46 and 4.61 times as large as their positive counterparts. When considering the complete set of percentiles between the median and the 99th, we in fact find that the maximum ratios of negative to positive treatment effect (i.e., how much larger the negative effect is than the equivalent positive effect) are 3.26 and 2.63 for views and replies, compared to maximum ratios of 6.27 and 6.14 for likes and reposts. The *minimum* ratios among these percentiles are in fact 4.46 and 3.01 for likes and reposts, compared to 0.934 and 0.818 for views and replies. These findings suggests that,

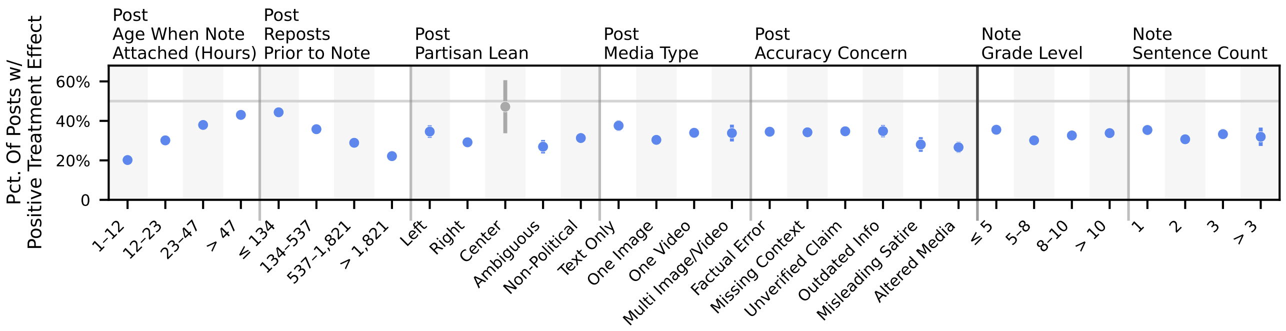

Fig. S2. Factors associated with positive treatment effects on reposts. Among all treated posts, the percentage with positive individual treatment effects after 48 hours, broken down by (i) hours from post creation to note attachment, (ii) number of reposts at the time of note attachment, (iii) post partisanship, (iv) number of images and videos, (v) accuracy concern raised by the community note writer, (vi) readability (Flesch–Kincaid grade level), and (vii) length of the community note.

beyond positive signals of engagement being more likely than non-positive signals to decrease due to note attachment, when they do decrease, they also tend to do so by a relatively larger amount.

Considering reposts, as we do in *Main Text, Fig. 4*, we find that many factors associated with differences in average treatment effects (e.g., the amount of time between post creation and note attachment) are also associated with posts' probabilities of having a positive treatment effect. In *Fig. S2*, we plot the percentage of treatment effects that are positive, disaggregated by variables previously found to be associated with differences in treatment effects. The age of a post when it receives a note has one of the stronger pairwise associations with whether attachment will lead to an increase in reposts: 20.2% of posts in the most rapid quartile (those noted within 12 hours of creation) have positive treatment effects, compared to 43.0% of posts in the final quartile (those noted after 47 hours). We discuss the heterogeneity of effects by popularity, i.e., number of reposts prior to note attachment in the next section. Partisan lean, media type, accuracy concern, grade level, and sentence count show similar, if less strong, relationships with the positivity of the reposts treatment effects as they do with its magnitude.

3. Heterogeneity in Effects on Reposts Based on Popularity Before Note Attachment

As noted in Section *Factors Associated with Large Effects* in the main text, we find that a post's popularity prior to receiving a community note is associated with the absolute treatment effect it receives. As in the main text, we center our analysis on reposts. We find that for posts with the fewest reposts prior to note attachment (i.e., those in the first quartile, having 134 or fewer reposts), the mean absolute treatment effect after 48 hours was a change of 1 repost (95% CI: [−10, 13]), or 1.4% overall. In comparison, the absolute changes for the second (134–537 reposts), third (537–1,821 reposts), and fourth (1,821+ reposts) quartiles were −67 (95% CI: [−79, −54]), −231 (95% CI: [−264, −198]), and −753 (95% CI: [−849, −657]), respectively, corresponding to percentage changes of −15.0%, −15.0%, and −10.9% overall.

The similarity in percentage changes between posts in the second, third, and fourth quartiles suggests that much of the difference in absolute changes (−67, −231, and −753) may be due simply to the number of users who see a post and can therefore be affected by note attachment. In other words, community notes on posts of a sufficiently large size have relatively similar effects on individual viewers' reposting behavior. However, community notes attached to posts in the smallest popularity quartile do appear to have a different aggregate effect on reposting behavior. As shown in *Fig. S2*, posts in this quartile are more likely to have notes lead to increases in reposts: 44.4% of posts in the bottom quartile have positive treatment effects, compared to 35.8%, 28.9%, and 22.2% of posts in the larger quartiles. These findings may indicate that attaching notes sometimes draws additional attention to low-visibility posts, resulting in a higher likelihood of positive changes. With that said, we also note that the coefficient of variation is much larger in magnitude for small posts: 190, compared to −3.92, −2.90, and −2.57 for posts in the larger quartiles. The large amount of relative variation in this quartile suggests that notes attached to low-visibility posts tend to have a less consistent effect than notes on more visible posts.

4. Factors Associated with Note Effectiveness on the Number of Views, Replies, and Likes

In addition to investigating factors associated with large effects on reposts, we also examine factors associated with large effects on views, replies, and likes. We plot conditional treatment effects on views, replies, and likes in *Fig. S3*, *Fig. S4*, and *Fig. S5*, respectively. Most factors associated with large effects on reposts tend to show similar associations with these additional metrics; however, we note some nuances below. First, considering the partisanship of the post, we find that the treatment effects on views, replies, and likes are all larger in magnitude for right-leaning posts than for left-leaning posts. This gap appears largest for replies and views, metrics that are not as clear a positive signal of agreement from a user as a reply or a like. In fact, we estimate positive (although not statistically significant) average treatment effects on views and replies for left-leaning posts. Another trend we observe is that while the treatment effect on reposts declines as notes become more readable, the association with views is in the opposite direction. The more readable a note is, the larger the effect it tends to have on a post's reposts, but the smaller the effect it tends to have on views. This observation supports the hypothesis that unclear notes may act as a warning signal, causing users to quickly move past a post. In contrast, more comprehensible notes may engage users, making them less likely to later express agreement with the post.

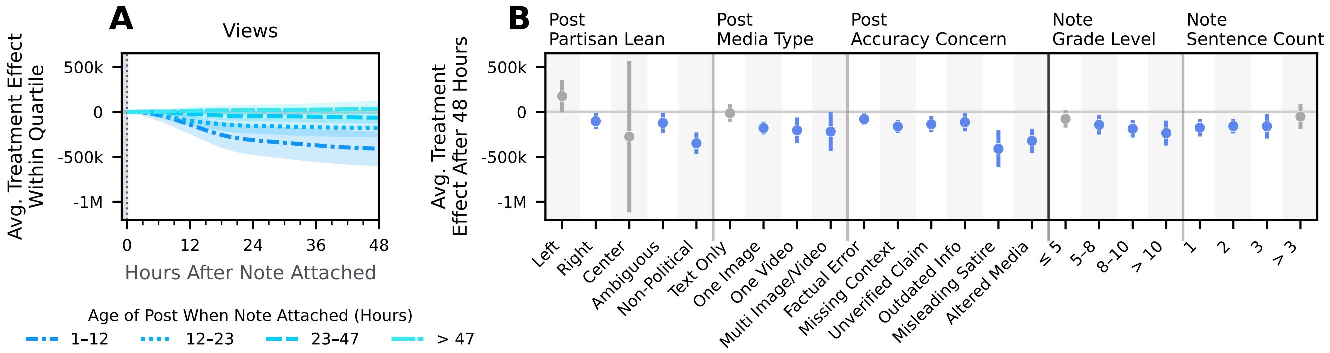

Fig. S3. Factors associated with effects on *views*. *(A)* Average treatment effects for treated posts, stratified by hours from post creation to first note attachment. Bins represent note speed quartiles. *(B)* Average treatment effects for noted posts after 48 hours, based on (i) the post's partisan lean, (ii) the number of images and videos, (iii) the accuracy concerns raised by the community note writer, (iv) the readability (Flesch–Kincaid grade level), and (v) the length of the community note. The error bands and error bars represent 95% confidence intervals.

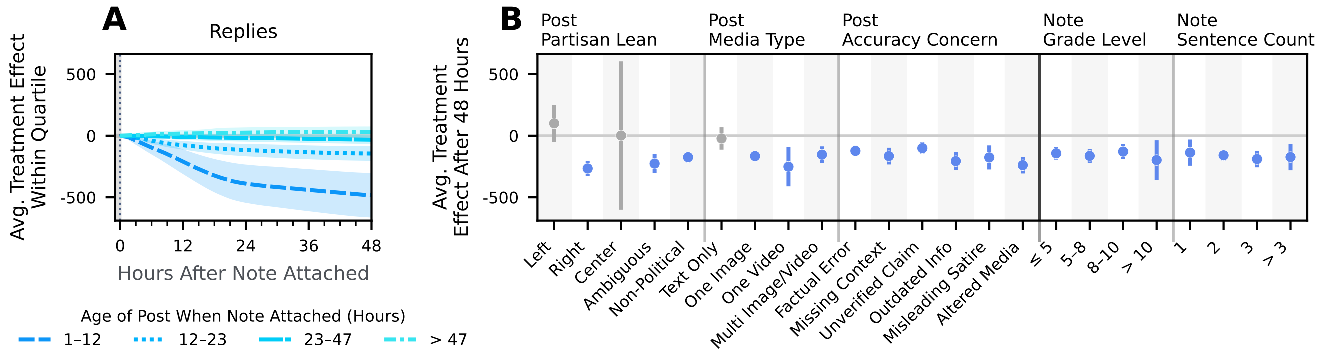

Fig. S4. Factors associated with effects on *replies*. *(A)* Average treatment effects for treated posts, stratified by hours from post creation to first note attachment. Bins represent note speed quartiles. *(B)* Average treatment effects for noted posts after 48 hours, based on (i) the post's partisan lean, (ii) the number of images and videos, (iii) the accuracy concerns raised by the community note writer, (iv) the readability (Flesch–Kincaid grade level), and (v) the length of the community note. The error bands and error bars represent 95% confidence intervals.

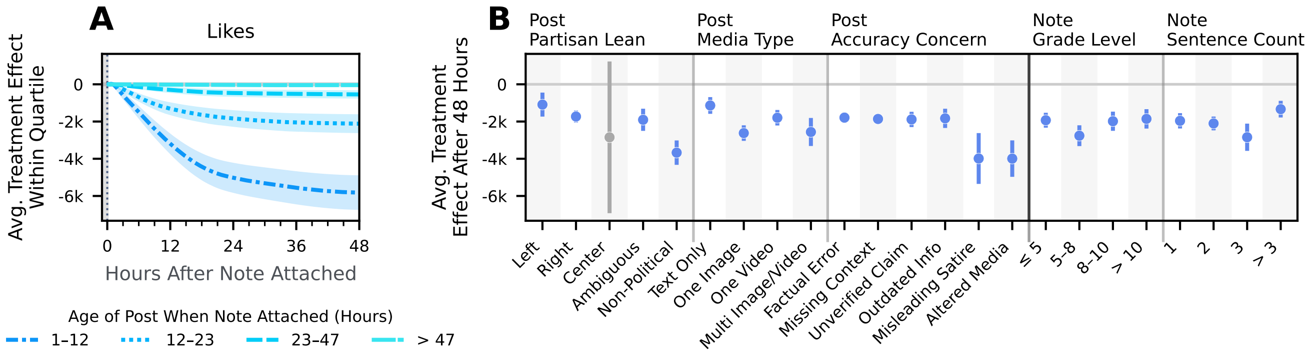

Fig. S5. Factors associated with effects on *likes*. *(A)* Average treatment effects for treated posts, stratified by hours from post creation to first note attachment. Bins represent note speed quartiles. *(B)* Average treatment effects for noted posts after 48 hours, based on (i) the post's partisan lean, (ii) the number of images and videos, (iii) the accuracy concerns raised by the community note writer, (iv) the readability (Flesch–Kincaid grade level), and (v) the length of the community note. The error bands and error bars represent 95% confidence intervals.

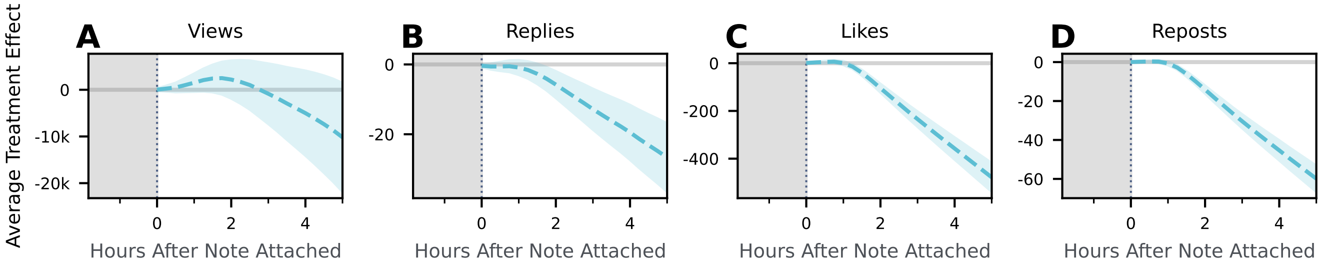

Fig. S6. Effects of note attachment on views, replies, likes, and reposts during the first five hours after a post's first note is classified as "helpful."

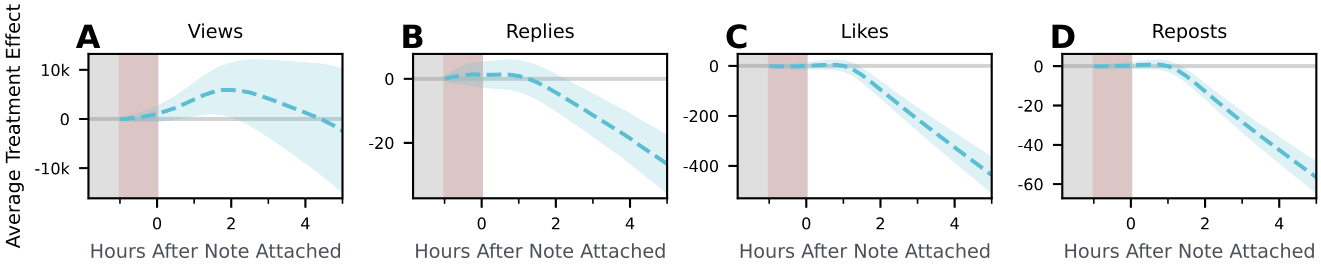

Fig. S7. One-hour placebo test: Shows the effects of note attachment on views, replies, likes, and reposts when using a one-hour backdate. The estimated effects at the end of the backdate period—highlighted in light red, when we would expect a null effect—are 1,028 views (95% CI: $[-341, 2, 397]$), 1.28 replies (95% CI: $[-2.36, 4.92]$), -0.154 likes (95% CI: $[-9.57, 9.26]$), and 0.287 reposts (95% CI: $[-0.839, 1.41]$). The slight initial increase and subsequent decrease in views is likely due to posts appearing in the "Rated Helpful" tab on the Community Notes webpage, which displays the most recently noted posts and is frequently visited by Community Notes volunteers.

5. In-Time Placebo Test

Traditional methods for performing statistical inference are difficult to adapt to Synthetic Control Methods, and as such, researchers typically validate synthetic control-based estimates using falsification experiments (4). Similar to A/A tests, falsification experiments involve altering the data such that no treatment effect should be observed, before rerunning the synthetic control construction to ensure that no effect is, in fact, estimated. One such exercise is the in-time placebo test (5), first introduced by Heckman and Hotz (6). This test involves "backdating" the treatment time, i.e., artificially considering it to have occurred earlier than it did, and then constructing the synthetic controls using only observations up until this backdated time. Any non-null treatment effects observed during the backdate period (i.e., between the backdate and the true treatment time) would suggest that the observed effects are not due to the treatment itself and would therefore undermine the credibility of the synthetic control-based treatment effect estimates.

Prior to October 23rd, 2023, there was a delay between when a note was classified as "helpful" and when it was displayed on the X platform (7). This delay arises for two reasons: (1) the timestamps indicating when a note achieved a "helpful status" in the publicly available data—which we use to determine the treatment time in our analysis—correspond to the start time of the Community Notes algorithm's computation, as documented in the open-source code, and (2) the time required for the algorithm's output to propagate through X's system and for the notes to appear on the platform. This delay typically lasts around one hour; however, there is unknown variation in its length. Therefore, we decided to consider the time when a post first received a "helpful" note recorded in the public Community Notes data as its treatment time. (For ease of exposition, we use the term "note attachment" to refer to the treatment time throughout our work, and differentiate between note attachment and treatment time only in this section.) Since the actual note attachment typically occurred about an hour after the treatment time used, our synthetic controls-based estimation of the treatment effects includes a natural in-time placebo test.

While not easily visible in *Main Text, Fig. 2* due to the axes' scales, we do find a null effect extending until approximately one hour after the first note was classified as "helpful." *Fig. S6* shows a subset of *Main Text, Fig. 2*, focusing on only the five hours following the first "helpful" note, rather than the full 48-hour period. As seen in the figure, we find that replies, likes, and reposts do not begin to decline meaningfully until about one hour after treatment, coinciding with the typical time when notes began to be displayed to users. The slight initial increase and subsequent decrease in views is likely due to posts appearing in the "Rated Helpful" tab on the Community Notes webpage, which displays the most recently noted posts and is frequently visited by Community Notes volunteers.

In addition to the natural in-time placebo test, we also perform an in-time placebo test with a one-hour backdate, estimating the synthetic controls using only observations up until one hour before a post received its first "helpful" note. As shown in *Fig. S7*, we again find that the treatment effect does not begin to decline until approximately one hour after treatment occurs. We also find that the confidence intervals estimated after backdating include zero for all metrics. One hour after the synthetic control estimation period ends (i.e., the time we consider the start of the treatment), the estimated effects were $1,028$ views (95% CI: $[-341, 2, 397]$), 1.28 replies (95% CI: $[-2.36, 4.92]$), -0.154 likes (95% CI: $[-9.57, 9.26]$), and 0.287 reposts (95% CI: $[-0.839, 1.41]$).

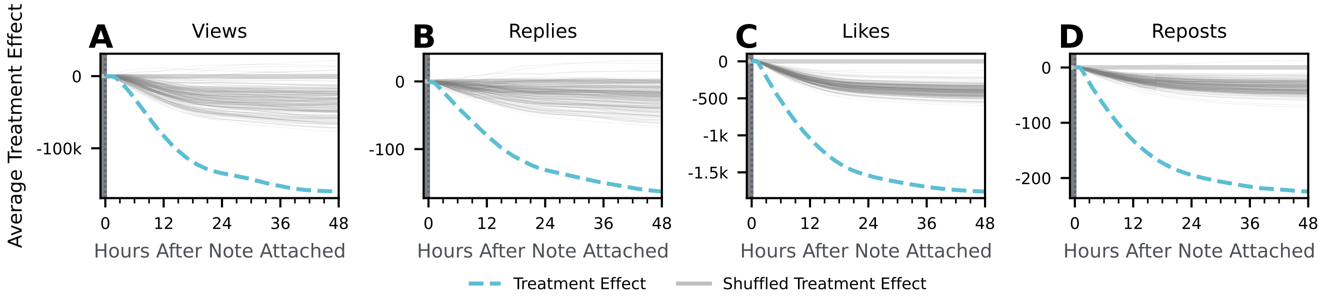

Fig. S8. Permutation test. Each gray line shows a "placebo" effect estimated from shuffling the treatment assignment (shuffling which set of posts, from both treatment and control, are considered to be in treatment) and then measuring an average treatment effect on this artificial treatment group. To avoid cluttering the plot, we show a random sample of 200 permutations. The dashed blue lines show the average treatment effect among the posts that actually received treatment.

6. Permutation Test

Permutation tests are another common test of validity used with Synthetic Control Methods (5, 8). Rather than testing whether a null effect is observed when artificially shifting the treatment time, as with in-time placebo tests, permutation tests artificially shuffle the treatment assignment. The complete set of units (both treatment and control) is pooled, and a random selection of the pooled units (equal in size to the original treatment group) is considered to be treated for the purposes of the artificial experiment. An average "placebo" effect is calculated for the artificially treated posts using all other posts as controls, regardless of whether they actually received treatment. This procedure is applied repeatedly with different samples of artificially treated posts, and a permutation distribution of average "placebo" effects estimated from these repetitions is compared to the observed average treatment effect. If the magnitude of the observed average treatment effect is extreme relative to the permutation distribution, it is deemed significant. A p-value is calculated by comparing the observed treatment effect to the average "placebo" effects found when shuffling the treatment assignment.

A typical permutation test accounts for two types of variability in the average treatment effect: variability due to which units receive treatment and variability due to which units are used as controls. Each unit considered to be treated is assumed to have a true, although unobserved, treatment effect. Treatment effects may differ across units, and a typical permutation test measures how much the average treatment effect changes when different units' treatment effects are included. This aspect of permutation testing follows logic similar to that of Fisher's exact test, comparing the observed outcome to the distribution of outcomes under random assignment. In addition to variability in the treated units, a permutation test also typically quantifies variability in the units used as controls. While each unit considered to be treated is assumed to have a true treatment effect, the synthetic control estimation process may not be able to estimate this effect precisely. By using different sets of control units in different iterations of a permutation test, one can quantify the extent to which variability in the estimation process leads to different average treatment effects on the treated units.

Permutation testing typically involves carrying out the complete treatment effect estimation process multiple times, including finding a synthetic control for each post considered to be treated in each permutation. Due to the size of our dataset, estimating average treatment effects for a single set of posts requires around one week, even with extensive parallelization across many processes, making a test involving even hundreds of iterations prohibitively long. As we are unable to perform a typical permutation test, we instead rely on the following procedure. For each unit in the pooled set, regardless of its true treatment status, we construct a synthetic control using all other units in the pooled set, including both treated and control units, as donors. The remainder of the effect estimation procedure is carried out as usual. Once treatment effects have been estimated for each unit, we then sample a set of units from the pooled set (of size equal to the number of truly treated units) and calculate an average "placebo" effect using these units. We perform multiple iterations of this sampling and compare the observed average treatment effect for the truly treated units to the distribution of average "placebo" effects estimated in these iterations. This computationally feasible procedure accounts for variability due to the units that receive treatment but does not account for variability due to the set of units used as controls.

Fig. S8 shows the distribution of average "placebo" effects obtained by permuting the treatment assignment vector and the observed average treatment effects for the truly treated units. The average "placebo" effects tend to be negative because, in each permutation, some of the truly treated units—which tend to have negative individual treatment effects—are reassigned as treated. The observed average treatment effects among the truly treated units (dashed blue lines) are smaller than those reported in the main text since, as described above, the treatment effects are estimated using both treatment and control units as donors, rather than just the control units. We perform 100,000 permutations, and for each metric, we find that the observed treatment effects are strictly larger in magnitude than all average "placebo" effects, resulting in p-values of 0.00001.

7. Synthetic Controls with Additional Post Content Embeddings

In the analyses reported in the main text, we constructed a synthetic control that closely resembles each treatment post's engagement (number of views, replies, likes, and reposts) and diffusion (repost cascade depth, breadth, and structural virality) trajectories prior to note attachment. While we restrict the donor pool for constructing the synthetic controls to posts suspected

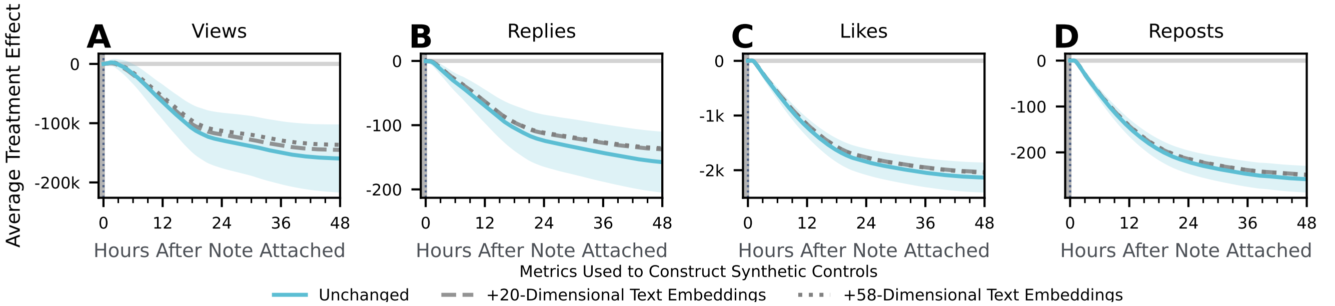

Fig. S9. Synthetic controls with additional post content embeddings. The grey lines show the estimated treatment effects when including 20- and 58-dimensional post text embeddings in the construction of the synthetic controls, in addition to the posts' pre-treatment engagement and diffusion trajectories. All estimates fall within the 95% confidence intervals of the original estimates based solely on the engagement and diffusion trajectories.

to be misleading and for which community notes have been proposed, one possible concern is that the posts which are given the largest weights in constructing the synthetic controls may be content-wise very different from the treatment post, e.g., they might be on more polarizing or controversial topics than the treatment post, which could make it harder for them to achieve the consensus required by the Community Notes algorithm for a note to be attached. To examine this concern, we perform robustness analyses that also take into account the semantic embeddings of the posts' content in addition to their diffusion and engagement trajectories.

To generate semantic embeddings of the posts, we use Google's Gemini `text-embedding-004` model with the task type set to "clustering," which produces 768-dimensional vectors per post. Before embedding the posts, we preprocess the text by removing any URLs, as most are shortened URLs that do not carry any semantic information. As the synthetic controls method involves minimizing the squared Euclidean distance between the synthetic control and treatment units, across all metrics considered, we use Principal Component Analysis (PCA) to reduce the dimensionality of the text embeddings, preventing them from dominating the distance relative to the engagement and diffusion metrics. We reduce the embeddings to two sizes: (i) 58 dimensions, considered optimal according to the method by Satopaa et al. (9) for locating a "knee" in the curve of proportion of variance explained by each component, and (ii) 20 dimensions, a slightly smaller size included for comparison. In our main analysis, before running the synthetic controls procedure, we scale each covariate by its standard deviation among the treated units; however, in this case, we standardize the PCA-reduced text embeddings by dividing each dimension by the mean standard deviation of all dimensions for the treated units. This standardization preserves the relative importance of each PCA component—as measured by the variance explained—while giving the embeddings, as a group, the same per-dimension weight as other covariates. Manually spot-checking the outputs of the synthetic controls procedure, we observe that, in most cases, the donor posts given the largest weights are about the same claim or the same type of misinformation (e.g., vaccine-related) as the treated post, suggesting that the inclusion of the text embedding has the desired effect.

When incorporating the post text embeddings with 20 and 58 dimensions into the construction of the synthetic controls, we estimate treatment effects on views 48 hours after note attachment of $-145,097$ and $-136,535$, respectively (*Fig. S9*). These estimates fall well within the 95% confidence interval for the average treatment effect on views when text embeddings are not included in the synthetic control construction, which was $-159,592$ (95% CI: $[-214,839, -104,344]$). Similarly, the effects on replies are -138 and -136, relative to the originally estimated effect of -158 (95% CI: $[-203, -112]$). For likes, the estimated treatment effects were $-2,033$ and $-2,043$, relative to the originally estimated effect of $-2,134$ (95% CI: $[-2,385, -1,884]$). Finally, for reposts, the estimated effects are -248 and -249, relative to the originally estimated effect of -259 (95% CI: $[-285, -232]$). These results suggest that the engagement and diffusion trajectories already capture the key information about the posts, and that incorporating post content in the construction of the synthetic controls does not significantly change the treatment effect estimates.

8. Synthetic Controls With Donor Pools Restricted to Control Posts with High-Scoring Notes

In the previous section, we expanded the set of covariates used to construct the synthetic controls by incorporating semantic embeddings of the posts' text, in addition to the pre-treatment engagement and diffusion trajectories. This addition allowed us to test whether ensuring that the synthetic controls are composed of control posts with similar content to the treatment post affects our treatment effect estimates. However, despite including a broad set of covariates when constructing the synthetic controls, observational causal inference inherently carries the risk that the control units may differ systematically from the treated units in unmeasured ways. As a result, the observed differences in outcomes between the two groups may reflect these unobserved confounders rather than the effect of the treatment itself.

In particular, the procedure by which community notes are selected to be shown on X creates one noteworthy way in which the treatment and control groups differ. A community note is shown on the platform only once it has been rated "helpful" by many users with diverse views and achieves a helpfulness score above 0.4. Therefore, it is plausible that the control posts, which never received "helpful" notes, do not contain the same distribution of content types as the treatment posts or otherwise differ in notable ways. As a result, they might not be a suitable pool from which to construct synthetic controls.

To test whether this discrepancy might affect our results, we limit the donor pool used to construct synthetic controls to

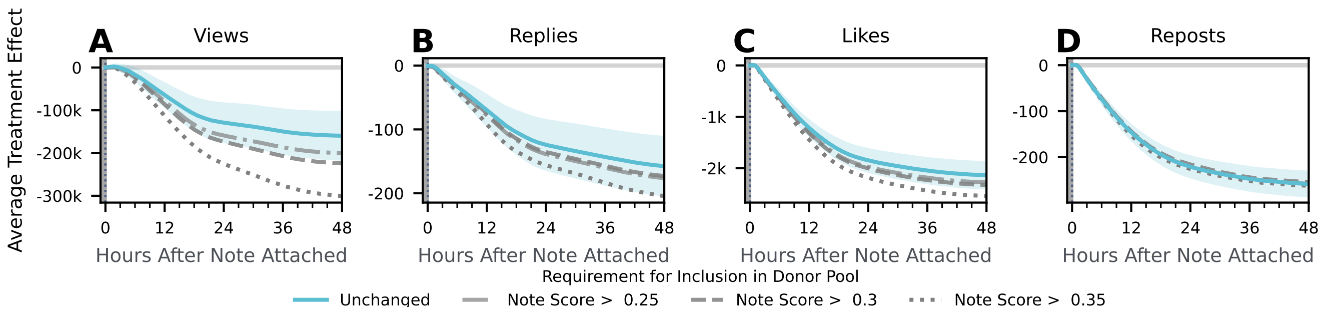

Fig. S10. Synthetic controls with donor pools restricted to control posts with high-scoring notes. The gray lines show the estimated treatment effects when limiting the donor pool to posts with notes that have helpfulness scores above 0.25, 0.3, and 0.35. The solid blue lines and the corresponding error bands show the original point estimates and 95% confidence intervals based on an unrestricted donor pool, as reported in the main text.

only those control posts that received a note whose helpfulness score exceeded 0.25, 0.3, and 0.35. By placing these restrictions, we seek to limit the donor pool to control posts most similar to those in treatment, in terms of the helpfulness of their notes. Since the helpfulness scores can change over time and notes may be deleted, we rerun the Community Notes algorithm using the publicly released data from X for each day of our study period and take the maximum score each note ever achieved. Applying these score thresholds reduces the size of the donor pool from 33,321 units to 8,760 units, 5,084 units, and 2,394 units, respectively.

When restricting the donor pool to units that receive a helpfulness score above 0.25, 0.3, and 0.35, we estimate treatment effects on views 48 hours after note attachment of −200,327, −224,108, and −300,596, respectively (*Fig. S10*). The estimated treatment effect when using the complete donor pool is −159,592 (95% CI: [−214,839, −104,344]). The effects on replies are −176, −173, and −204, relative to the originally estimated effect of −158 (95% CI: [−203, −112]). For likes, the estimated treatment effects are −2,275, −2,325, and −2,541, relative to the originally estimated effect of −2,134 (95% CI: [−2,385, −1,884]). Finally, for reposts, the estimated effects are −258, −254, and −262, relative to the originally estimated effect of −259 (95% CI: [−285, −232]).

The larger treatment effects on views found when restricting the donor pool suggest that our original results may be overestimates rather than underestimates. However, we give a note of caution toward this interpretation. We find that using a restricted donor pool leads to larger differences between the treatment units and their synthetic controls in terms of the pre-treatment engagement and diffusion metrics. This discrepancy indicates that the smaller donor pool results in lower-quality matches, which may lead to biased estimates. Thus, we focus our presentation on the results using the full donor pool, as presented in the main text, and consider the analysis based on restricted donor pools as a robustness check.

References

1. S Goel, A Anderson, J Hofman, DJ Watts, The structural virality of online diffusion. *Manag. Sci.* **62**, 180–196 (2016).
2. S Vosoughi, D Roy, S Aral, The spread of true and false news online. *Science* **359**, 1146–1151 (2018).
3. H Wiener, Structural determination of paraffin boiling points. *J. Am. chemical society* **69**, 17–20 (1947).
4. A Abadie, A Diamond, J Hainmueller, Comparative politics and the synthetic control method. *Am. J. Polit. Sci.* **59**, 495–510 (2015).
5. A Abadie, Using synthetic controls: Feasability, data requirements, and methodological aspects. *J. Econ. Lit.* **59**, 391–425 (2021).
6. JJ Heckman, VJ Hotz, Choosing among alternative nonexperimental methods for estimating the impact of social programs: The case of manpower training. *J. Am. Stat. Assoc.* **84**, 862–874 (1989).
7. X, Note ranking algorithm (2023) https://communitynotes.x.com/guide/en/under-the-hood/ranking-notes. Accessed 20 August 2025.
8. A Abadie, J L'Hour, A penalized synthetic control estimator for disaggregated data. *J. Am. Stat. Assoc.* **116**, 1817–1834 (2021).
9. V Satopaa, J Albrecht, D Irwin, B Raghavan, Finding a "kneedle"' in a haystack: Detecting knee points in system behavior in *International Conference on Distributed Computing Systems Workshops.* (IEEE), pp. 166–171 (2011).
